# Supplementary figures and images for: Exploring T. cruzi IMPDH as a promising target through Chagas Box screening and AVN-944 inhibition
Source: Antimicrob Agents Chemother. 2026 Jan 22;70(3):e01210-25. doi: 10.1128/aac.01210-25 (PMC12959148; doi:10.1128/aac.01210-25)

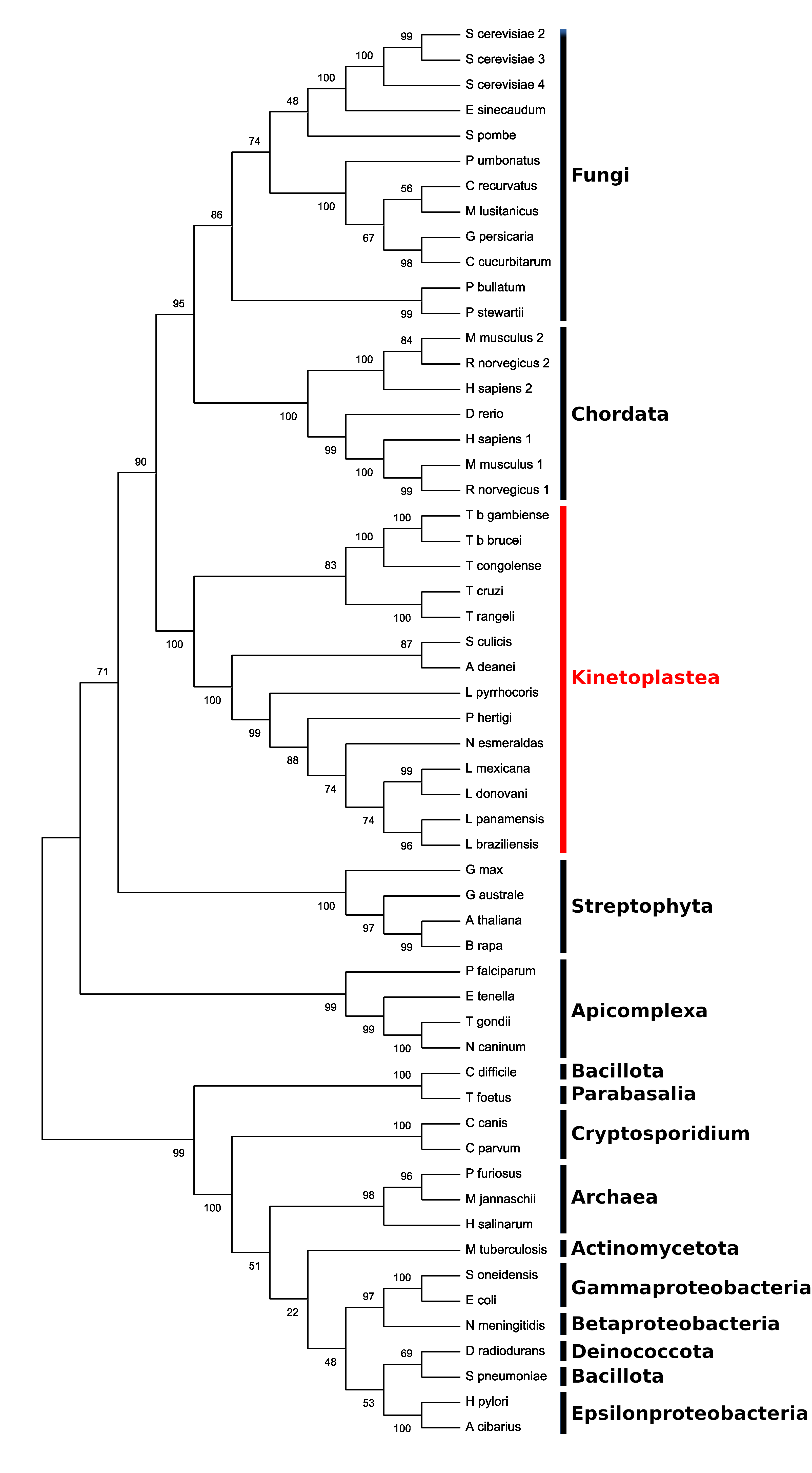

Supplement: Fig. S1 — Phylogenetic analysis of inosine monophosphate dehydrogenases from 56 different taxa. [file aac.01210-25-s0001.tif]
