# Supplementary material for: Exploring T. cruzi IMPDH as a promising target through Chagas Box screening and AVN-944 inhibition
Source: Antimicrob Agents Chemother. 2026 Jan 22;70(3):e01210-25. doi: 10.1128/aac.01210-25 (PMC12959148; doi:10.1128/aac.01210-25)
Supplement: Supplemental material — Supplemental figure captions. [file aac.01210-25-s0004.docx]

**Supplementary figure captions**

**Fig. S1. Phylogenetic analysis of inosine monophosphate dehydrogenases from 56 different taxa.** The phylogenetic tree was inferred with 56 IMPDH amino acid sequences using the Neighbor-Joining method [1]. The optimal tree with the sum of branch length = 9,232 is shown. The percentage of replicate trees in which the associated taxa clustered together in the bootstrap test (5.000 replicates) are shown next to the branches [2]. The evolutionary distances were computed using the Poisson correction method [3] and are in the units of the number of amino acid substitutions per site. The pairwise deletion option was applied to all ambiguous positions for each sequence pair resulting in a final data set comprising 760 positions. The following sequences listed with their NCBI accession code were used: *S. cerevisiae* 2 (NP_012088.3)*, S. cerevisiae* 3 (NP_013536.3)*, S. cerevisiae* 4 (NP_013656.1)*, E. sinecaudum* (XP_017989130.1)*, S. pombe* (NP_595702.1)*, P. umbonatus* (KAI8986466.1)*, C. recurvatus* (XP_051385700.1)*, M. lusitanicus* (KAF1806276.1)*, G. persicaria* (XP_051435643.1)*, C. cucurbitarum* (KAI8361798.1)*, P. bullatum* (KAJ3116508.1)*, P. stewartii* (KAL2918580.1)*, M. musculus* 1 (NP_001289862.1)*, M. musculus* 2 (NP_035960.2)*, R. norvegicus* 1 (NP_001385786.1)*, R. norvegicus* 2 (NP_954530.2)*, H. sapiens* 1 (NP_001136045.1)*, H. sapiens* 2 (NP_000875.2)*, D. rerio* (NP_001002177.1)*, T. b. gambiense* (XP_011779130.1)*, T. b. brucei* (P50098.1)*, T. congolense* (BAT33662.1)*, T. cruzi* (XP_805772.1)*, T. rangeli* (ESL06509.1)*, S. culicis* (EPY26926.1)*, A. deanei* (EPY43134.1)*, L. pyrrhocoris* (XP_015661374.1)*, P hertigi* (XP_067757840.1)*, N. esmeraldas* (KAK7197932.1)*, L. mexicana* (XP_003874334.1)*, L. donovani* (TPP50911.1)*, L. panamensis* (XP_010698138.1)*, L. braziliensis* (XP_001564275.1)*, G. max* (XP_003532912.3)*, G. australe* (KAA3485993.1)*, A. thaliana* (NP_178065.1)*, B. rapa* (XOT41424.1)*, P. falciparum* (XP_001352079.1)*, E. tenella* (XP_013230284.1)*, T. gondii* (Q4VRV8.1)*, N. caninum* (XP_003883536.1)*, C. difficile* (WP_003423106.1)*, T. foetus* (XP_068358496.1)*, C. canis* (KAJ1609978.1)*, C. parvum* (AAL83208.1)*, P. furiosus* (AAL80409.1)*, M. jannaschii* (Q59011.1)*, H. salinarum* (Q9HQU4.2)*, M. tuberculosis* (P9WKI7.1)*, S. oneidensis* (WP_011073172.1)*, E. coli* (P0ADG7.1)*, N. meningitidis* (WP_002246200.1)*, D. radiodurans* (WP_010888513.1)*, S. pneumoniae* (WP_000073427.1)*, H. pylori* (Q9ZL14.1)*, A. cibarius* (QKJ27026.1)*.* Evolutionary analyses were conducted in MEGA12 [4] utilizing up to 3 parallel computing threads.

**Fig. S2. Multiple sequence alignment of IMPDH enzymes highlighting conserved catalytic and regulatory motifs.** Amino acid sequences of IMPDHs from selected prokaryotic and eukaryotic organisms were aligned using Clustal Omega and visualized with ESPript 3, specifically the sequences used were: *Tritrichomonas foetus* (XP_068358496.1), *Trypanosoma cruzi* (XP_805772.1), *Trypanosoma brucei* (AAB46420.1), *Leishmania donovani* (XP_003860332.1), *Homo sapiens* 1 (NP_001136045.1), *Homo sapiens* 2 (NP_000875.2), *Mus musculus* 1 (NP_001289862.1), *Mus musculus* 2 (NP_035960.2), *Plasmodium falciparum* (XP_001352079.1), *Arabidopsis thaliana* (NP_178065.1), *Escherichia coli* (P0ADG7.1). Residues involved in XMP/IMP binding (e.g., S323, D358, M408, G409, Q435 in *T. cruzi* IMPDH) and NAD⁺ binding (e.g., G320, D268, S269, S270, R247, Y276) are indicated with blue and red arrows, respectively. Sites identified in *T. brucei* IMPDH (*Tb*IMPDH) that coordinate regulatory ligands are also indicated: GMP-binding residues are indicated with purple arrows (K115, S136, G137) and ATP-binding residues with orange arrows (S136, T156, K157, D158, T174, T180, H200, Y202, R219), all of which are conserved in *T. cruzi* IMPDH (*Tc*IMPDH), except for a single conservative S→T substitution at position 156. Peroxisomal targeting signal type 1 (PTS1) motifs are light-blue underlined, marking the SKL tripeptide conserved in trypanosomatid IMPDHs.

**Fig. S3.** **Michaelis–Menten plots illustrating the hyperbolic kinetics of *Tc*IMPDH activity with respect to its two substrates**. **(A)** Reaction velocity as a function of NAD⁺ concentration. **(B)** Reaction velocity as a function of IMP concentration. Error bars represent the standard deviation of reaction velocity for each substrate concentration. IMPDH activity was measured as described in Section 2.4 of the Materials and Methods in the main text.

**Supplementary references**

1. Saitou N. and Nei M. (1987). The neighbor-joining method: A new method for reconstructing phylogenetic trees. Molecular Biology and Evolution 4:406-425.

2. Felsenstein J. (1985). Confidence limits on phylogenies: An approach using the bootstrap. Evolution 39:783-791.

3. Zuckerkandl E. and Pauling L. (1965). Evolutionary divergence and convergence in proteins. Edited in Evolving Genes and Proteins by V. Bryson and H.J. Vogel, pp. 97-166. Academic Press, New York.

4. Kumar S., Stecher G., Suleski M., Sanderford M., Sharma S., and Tamura K. (2024). Molecular Evolutionary Genetics Analysis Version 12 for adaptive and green computing. Molecular Biology and Evolution 41:1-9.
